# Editorial: Multimodal brain image fusion: Methods, evaluations, and applications

**DOI:** 10.3389/fnins.2022.1128938

**Published:** 2023-01-06

**Authors:** Yu Liu, Jiayi Ma, Qiang Zhang, Wei Wei, Xun Chen, Zheng Liu

**Affiliations:** ^1^Department of Biomedical Engineering, Hefei University of Technology, Hefei, China; ^2^Electronic Information School, Wuhan University, Wuhan, China; ^3^School of Mechano-Electronic Engineering, Xidian University, Xi'an, China; ^4^Department of Radiology, The First Affiliated Hospital of University of Science and Technology of China, Hefei, China; ^5^Department of Neurosurgery, The First Affiliated Hospital of University of Science and Technology of China, Hefei, China; ^6^School of Engineering, University of British Columbia, Kelowna, BC, Canada

**Keywords:** multimodal medical images, image registration, image fusion, quality assessment, pattern recognition, deep learning

Multimodal medical imaging is playing an increasingly critical role in the diagnosis and treatment of various brain diseases like glioma, Alzheimer, ischemic stroke, epilepsy, etc. Medical images with different modalities such as computed tomography (CT), magnetic resonance imaging (MRI), and positron emission tomography (PET) focus on different categories of pathological information. Medical image fusion aims to combine the complementary information captured by different imaging modalities for better disease diagnosis and treatment. In recent years, medical image fusion has emerged as a very active topic with various fusion methods being proposed. In addition, the performance evaluation and downstream applications of medical image fusion are also attracting more and more attention. This Research Topic focuses on reporting advanced studies related to multimodal brain image fusion, including image fusion methods, objective evaluation approaches and specific applications in clinical problems. Twelve of the 16 articles submitted to this Research Topic were accepted for publication after a thorough peer-review process. A summary of the key research findings of these works is provided from three aspects as below.

## Multimodal brain image registration, fusion and fusion quality evaluation

Image registration is the prerequisite of many medical image processing tasks such as fusion and segmentation. Wang J. et al. proposed a medical image registration method based on the bounded generalized Gaussian mixture model (BGGMM), which can thoroughly describe the joint intensity vector distribution of pixels and highlight image details. The mixture model is formulated based on a maximum likelihood framework, and is solved by an expectation-maximization algorithm. With regard to image fusion methods, Wang A. et al. presented a disentangled representation-based multimodal brain image fusion method *via* group lasso penalty using an auto-encoder-based deep learning framework, aiming to fully exploit the redundancy and complement prior relationships among multimodal source images. A complementary group lasso penalty was designed to promote the disentanglement ability and ensure the complementary feature maps of significant modality information. This study demonstrated that the disentangled representation can improve the interpretability of feature representation, leading to better fusion quality. Zhang et al. proposed a local extreme map guided multimodal brain image fusion method to improve the feature extraction ability of the guided image filter. By iteratively applying this local extreme map guided image filter, the proposed method can extract multiple scales of bright and dark features from the multimodal brain images, and integrate these salient features into one informative fused image. In addition, the proposed scheme can be incorporated with various guided filters or other similar filters in pursuit of improving their feature extraction ability. In comparison to the great attention paid to the study of image fusion methods, few works have explored dedicated quality assessment approaches for medical image fusion. To address this issue, Tang et al. proposed a novel quality assessment method for medial image fusion based on the conditional generative adversarial networks by adopting the mean opinion scores (MOS) of the radiologists as the guiding condition. They demonstrated that their proposed method outperforms several commonly-used quality assessment metrics of image fusion, with excellent agreement with subjective evaluations.

## Applications of multimodal brain image fusion

Multimodal medical image fusion has been verified to be of great significance in various related high-level vision tasks such as classification and segmentation. Yi et al. proposed a multimodal classification architecture for the severity diagnosis of glaucoma. The proposed method integrates fundus images and gray scale images of the visual field as the input of the classification model. In addition, they introduced a plug-and-play classifier that adopts the Vision Transformer to extract the global dependencies of images, leading to improved accuracy of the diagnostic task. Li et al. conducted a study to investigate the stage of bi-modal fusion based on EEG and fNIRS for the classification task in hybrid brain-computer interfaces (BCIs). A Y-shaped neural network that fuses the bi-modal information in different stages was proposed. This study demonstrated that the early-stage fusion of EEG and fNIRS have significantly higher performance compared to middle-stage fusion and late-stage fusion. Liu et al. introduced both pixel-level and feature-level medical image fusion techniques for brain tumor segmentation, aiming to achieve more sufficient utilization of multimodal information. They presented a convolutional neural network (CNN)-based 3D pixel-level image fusion network to enrich the input modalities of the segmentation model and designed an attention-based feature fusion module for multimodal feature refinement. Xu et al. proposed a hybrid feature extraction network for medical image segmentation based on CNNs and Transformer. The proposed network can integrate the advantages of Transformer in capturing global contextual information and CNNs in extracting local features. Additionally, a multi-dimensional statistical feature extraction module was designed to strengthen low-dimensional texture features and enhance the segmentation performance. Tian et al. presented a method to combine light sheet microscopy (LSM) data with magnetic resonance histology (MRH) of the same specimen, with the aim of restoring the morphology of the LSM images to the in-skull geometry. They developed an image processing pipeline to restore the correct brain morphology of 3-dimensional cleared or stained mouse brain by registering the cleared brain data to MRH of the same specimen. Peng et al. introduced the minimally invasive puncture and drainage (MIPD) surgery using mixed reality holographic navigation technology (MRHNT) *via* integrating the holographic image and the real head. By wearing mixed reality holographic equipment, the precise location of intracranial hematomas, tumors, ventricles, and other structures with the perspective function can be understood, laying a theoretical foundation for implementation in neurosurgery.

## Joint analysis of multimodal data

Mononen et al. conducted a study to evaluate the variability among tasks of magnetoencephalography (MEG)-functional magnetic resonance imaging (fMRI) relationship using data recorded during three distinct naming tasks from the same set of participants. The results demonstrated that the MEG-fMRI correlation pattern varies according to the performed task. In addition, the electromagnetic-hemodynamic correlation could serve as a more sensitive proxy for task-dependent neural engagement in cognitive tasks than isolated within-modality measures. Gallego-Rudolf et al. characterized the impact of the ballistocardiographic (BCG) artifact on resting-state EEG spectral properties and compared the effectiveness of seven common BCG correction methods to preserve EEG spectral features. They also assessed if these methods retained posterior alpha power reactivity to an eyes closure-opening task and compared the results from EEG-informed fMRI analysis using different BCG correction approaches.

## Author contributions

All authors listed have made a substantial, direct, and intellectual contribution to the work and approved it for publication.

